# Regulation of the regulators: Transcription factors controlling biosynthesis of plant secondary metabolites during biotic stresses and their regulation by miRNAs

**DOI:** 10.3389/fpls.2023.1126567

**Published:** 2023-03-02

**Authors:** Mohini Kajla, Amit Roy, Indrakant K. Singh, Archana Singh

**Affiliations:** ^1^ Department of Botany, Hansraj College, University of Delhi, Delhi, India; ^2^ Excellent Team for Mitigation (ETM), Faculty of Forestry and Wood Sciences, Czech University of Life Sciences Prague, Prague, Czechia; ^3^ Department of Zoology, Deshbandhu College, University of Delhi, New Delhi, India; ^4^ Jagdish Chandra Bose Center for Plant Genomics, Hansraj College, University of Delhi, Delhi, India; ^5^ Delhi School of Climate Change and Sustainability, Institution of Eminence, Maharishi Karnad Bhawan, University of Delhi, Delhi, India

**Keywords:** pest attack, pathogen infection, transcription factors, miRNAs, plant specialized/secondary metabolites

## Abstract

Biotic stresses threaten to destabilize global food security and cause major losses to crop yield worldwide. In response to pest and pathogen attacks, plants trigger many adaptive cellular, morphological, physiological, and metabolic changes. One of the crucial stress-induced adaptive responses is the synthesis and accumulation of plant secondary metabolites (PSMs). PSMs mitigate the adverse effects of stress by maintaining the normal physiological and metabolic functioning of the plants, thereby providing stress tolerance. This differential production of PSMs is tightly orchestrated by master regulatory elements, Transcription factors (TFs) express differentially or undergo transcriptional and translational modifications during stress conditions and influence the production of PSMs. Amongst others, microRNAs, a class of small, non-coding RNA molecules that regulate gene expression post-transcriptionally, also play a vital role in controlling the expression of many such TFs. The present review summarizes the role of stress-inducible TFs in synthesizing and accumulating secondary metabolites and also highlights how miRNAs fine-tune the differential expression of various stress-responsive transcription factors during biotic stress.

## 1 Introduction

Being sedentary, plants are continually defied by various environmental stresses, orchestrated into declination of their growth and yield. Biotic stresses include infection by various organisms such as bacteria, fungi, nematodes, viruses, and herbivory by insect pests. Plants continue to live with these stresses, decreasing productivity and yield loss (approximately 35% loss due to biotic stress) (Foyer et al., 2016). During 2020, due to decreased plant productivity, the United Nations (UN) has decided to acknowledge the year as an International year of plant health to motivate research in the area of plant science (Roux and Ham, 2020). It has also been estimated that food production will have to be increased by approximately 60% to feed the growing population of estimated 10 billion people. The primary reason for this loss in yield is increased pathogens and pests encountering crop plants due to abrupt climate change Jha et al., 2022. Plants have progressed numerous responses to shield themselves and counter-attacks of far-ranging pests and pathogens (Brading et al., 2000). Thus, plants tend to foray into a balance in their response and existential methods against biotic stress, which show deleterious effects (Iqbal et al., 2021; Kumar et al., 2020). The plant defensive response affecting the mechanisms at the molecular level has been explained profoundly (Cheng et al., 2012; Wang and Wang, 2019). However, the reasons for this diversity in metabolite production during biotic stress are not abundant.

An array of diverse metabolites is produced by plants, which can be either vital (primary metabolites) or non-vital (secondary or specialized metabolites), affecting the fundamental processes of growth and development (Obata, 2019). Plant Secondary metabolites (PSMs) are multifunctional metabolites, characteristically intricate in plant defenses, and environmental communication, especially during stress response. Plants’ diversified machinery for plant defense not only allow them to survive against stressors but also influence PSM accumulation (Pagare et al., 2015). The Biosynthesis of PSMs in response to stressful environments is controlled at the transcriptome level by numerous genes and transcription factors (TFs). TFs are sequence-specific DNA binding proteins that identify and bind specifically to the cis-regulatory sequences of the promoter regions of the targeted genes which may activate or repress their expression levels in response to developmental and other environmental cues (Patra et al., 2013). TFs in plants encode up to 10% of the total genes at diverse stages, thereby regulating signal-mediated gene expression. TF families, namely WRKY, MYB, NAC, and AP2/ERF, are crucial regulators of various genes, which contribute to the model choice for genetic engineering to boost the immunity of plants against diverse stress stipulations (Baillo et al., 2019).

During pest and pathogenic attacks, the concentration of PSMs alters due to the differential activity of TFs. miRNAs are one of the prime regulators which control the activity of TFs at the post-transcriptional level. Small, non-coding riboregulators known as miRNAs (20–24 nucleotides) also regulate eukaryotic gene expression through base pairing the complementary mRNA. The miRNA genes are transcribed by RNA polymerase II to produce an imperfect hairpin structure called pri-miRNAs. These pri-miRNAs are further processed by DICER-LIKE 1 (DCL1) to generate 70-100 nucleotides long hairpin structures called pre-miRNAs. The same DCL1 converts pre-miRNAs to mature miRNAs following their loading into the RISC complex, these mature miRNAs bind to mRNAs to cleave and silence the genes (Jones-Rhoades et al., 2006). miRNAs are recognized not only to regulate various plant processes related to their growth and development but also to control biosynthesis and accumulation of secondary metabolites during biotic stress (Bulgakov and Avramenko, 2015).

The investigation on the role of miRNA’s in regulation of transcription factors during biotic and abiotic stress has been extensively studied (Javed et al., 2020; Šečić et al., 2021). There are reported studies of various transcription factors involved in the regulation of synthesis and accumulation of PSMs (Pagano et al., 2021; Jan et al., 2021b). However, in this article, we have updated knowledge of the present understanding of TFs responsible for regulating plant secondary metabolites on biotic stress. We have also emphasized how these TFs are regulated by regulators such as miRNAs, which are directing gene expression at the post-transcriptional and translational levels. The production of secondary metabolites by maneuvering the role of miRNA in various economically important crops may improve the profitability of agriculture and food industries.

## 2 Plant secondary metabolites

Plants produce thousands of specialized organic compounds, divided conventionally into two broad forms, *i.e.*, primary and secondary metabolites (Obata, 2019). Primary metabolites portray a significant role in growth and development. In contrast, secondary metabolites are structurally diverse compounds that have been reported to be involved in plant defense (Pagare et al., 2015). They are known to contribute to the survival and health of plants and play a pivotal role in protecting them against abiotic (UV light, temperature, heavy metals, drought, salinity, etc.) as well as biotic stresses (herbivores, phytopathogens, fungi, bacteria, etc.) (Tiwari and Rana, 2015). It has been well-established that they show pharmaceutical properties (Verpoorte, 1998). Besides, it has also been reported that PSMs have been used commercially as agrochemicals in flavoring, fragrance, biopesticides, dyes, and food additives.

According to the studies on biochemical structure composition, PSMs have been broadly classified into two sets: nitrogen-containing (majorly alkaloids) and non-nitrogen-containing molecules (terpenes and phenols) (Patra et al., 2013). Plant-specialized metabolites (PSM) are chemically diverse in nature. The precursors for their biosynthesis are generated by either glycolysis or shikimic acid pathways, eventually leading to diversification based on cell types and environmental factors (Costa et al., 2012). PSMs have a much-limited presence in the plant kingdom. It has been noticed that they are recurrently found either in only one plant species or a taxonomically related genus (Jamwal et al., 2018). More than 100,000 SMs are produced in plants through different pathways, and their quality and quantity are mostly influenced by temperature and other biotic and abiotic factors (Meena et al., 2017).

**Figure 1:**
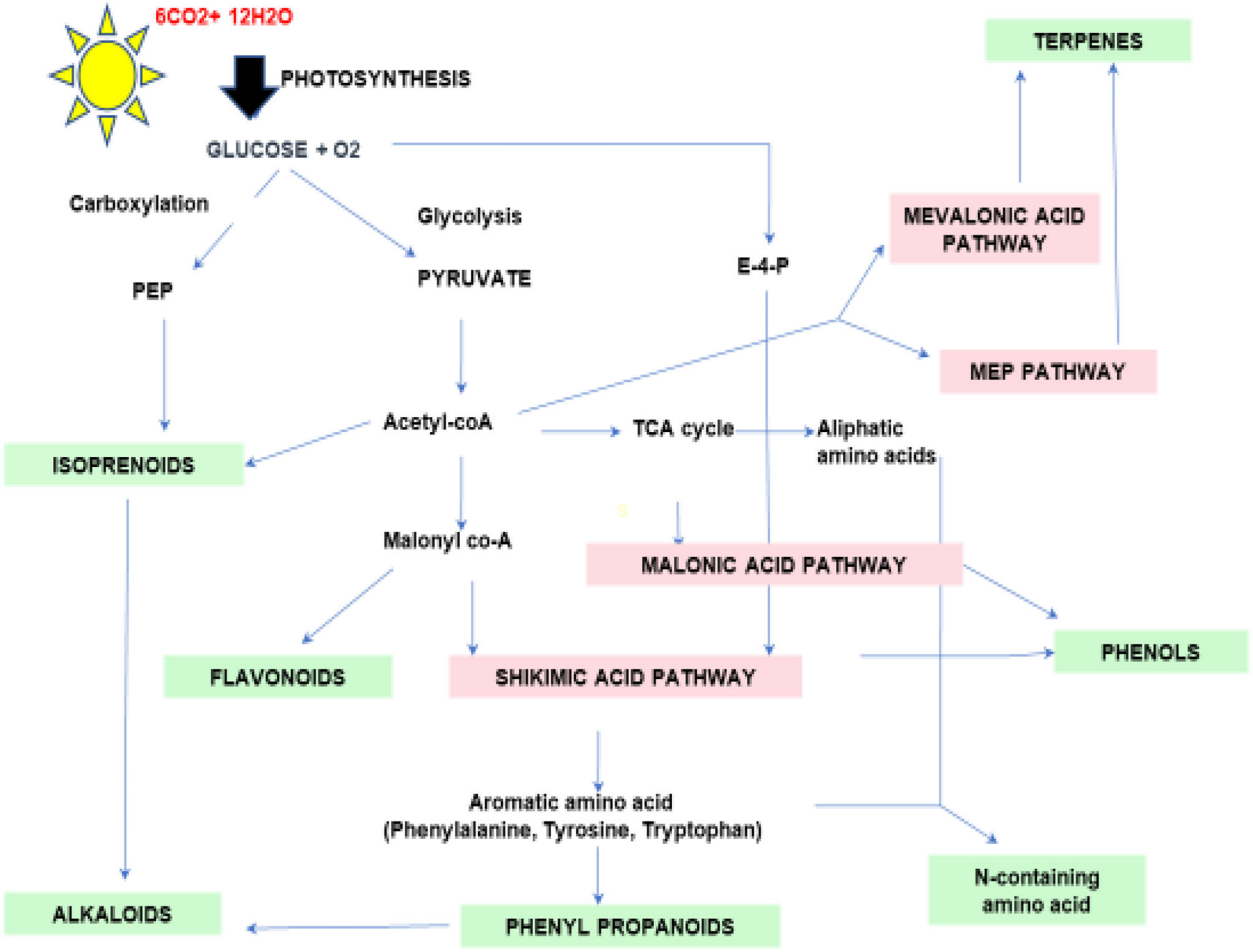
Schematic diagram of secondary metabolic biosynthesis pathway. Phosphenol pyruvate (PEP), Erythrose-4-phosphate (E-4-P), and Tricarboxylic acid (TCA) cycle.

Terpenes are the most diverse known PSMs synthesized from acetyl-CoA and glycolytic intermediates. They are formed by the fusion of 5 carbon units named isoprene and have a branched backbone. Terpenes consist of monoterpenes, diterpenes, sesquiterpene, triterpenes, tetraterpenes, and polyterpenes, which have significant importance in defense against pathogens and herbivory. Its biosynthesis includes two major pathways: the mevalonic acid pathway & methylerythritol phosphate pathway (MEP Pathway), which occur in plastids and are known to produce both isopentenyl diphosphate (IPP) and dimethylallyl diphosphate (DMAPP), respectively, which are the basic unit of terpene synthesis (**Figure 1**) (Khare et al., 2020). Some terpenes are identified as hormones (*e.g.*, gibberellins, a diterpene; brassinosteroids, triterpenes) and have crucial roles in growth and development. Terpenes, such as limonene and menthol, defend against herbivores (Ahmed et al., 2017; Lin et al., 2017). Another example is abietic acid, a class of diterpenes obtained from leguminous plants and pine trees that exhibit an antipathogenic effect (Lewis et al., 1997).

**Figure 2:**
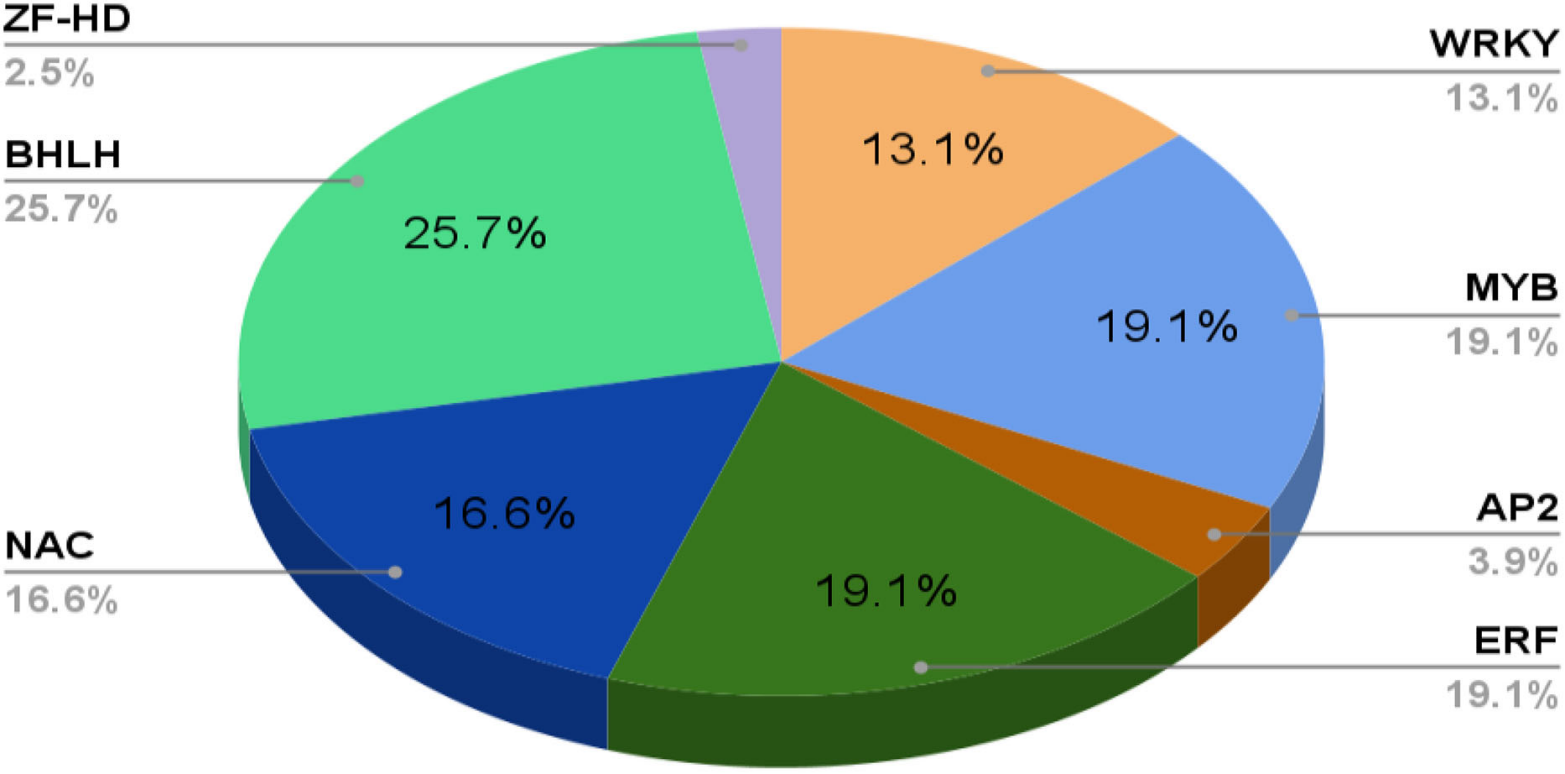
Schematic representation of the general pathways leading to various pathways of secondary metabolites biosynthesis such as phenols, terpenoids and N- containing secondary meabolites. PEP: Phosphoenol pyruvate, DMAPP : Dimethylallyl pyrophosphate, GPP: Geranyl pyrophosphate, FPP: Farnesyl pyrophosphate, GGPP: Geranylgeranyl diphosphate PAL : Phenylalanine ammonia-lyase, C4H:cinnamate-4-hydroxylase.

Phenolics are ubiquitous compounds with a hydroxyl group attached to an aromatic ring. It can be simple or complex; simple phenolics such as, (gallic acid, caffeic acid, *etc.*) and polyphenols (stilbenes, flavonoids, *etc.)* have several curative properties (Jan et al., 2021b). They have significantly evolved the modern medicine system, be it cancer or major diseases by showing properties like anti-inflammation, anti-cancerous, and many more. Besides, it also shows antioxidative properties against oxidative damage in plants due to harmful UV rays. In addition, the plant’s pigmentation of flowers and leaves is due to a complex phenol molecule named anthocyanin. Flavonoids, however, play an essential role in pollination to attract pollinators in exchange for rewards such as nectar and seed dispersal (Winkel, 2004; Crozier et al., 2006; Butelli et al., 2008). There are two major biosynthetic pathways: shikimic acid and malonic acid pathway, where phenylalanine ammonia-lyase (PAL) and chalcone synthase (CHS) are the key enzymes involved in the phenolic compound synthesis, which is produced under various stress conditions (Khare et al., 2020) (**Figure 2**). Flavonoids are the most common class of secondary metabolites that can be found in edible plant parts like grains, fruits, and vegetables. The phenylpropanoid pathway is the synthesis pathway and are derivatives of 2-phenylbenzyl-pyrone (Roy et al., 2022; Tajammal et al., 2022). Among flavonoids, luteolin, apigenin, quercetin, kaempferol, and many more have been isolated and reported to exhibit defense against pathogens, fungi, and other biotic stress (Kumar and Pandey, 2013; Li et al., 2019; Hassani et al., 2020).

Nitrogen-containing PSMs contain nitrogen molecules in the structure, and precursors include amino acids such as lysine, tyrosine, tryptophan, etc. It mainly includes alkaloids, cyanogenic glucosides & non-protein amino acids. Alkaloids such as morphine, berberine, vinblastine, and scopolamine have pharmaceutical properties, whereas cocaine, caffeine, and nicotine have sedative and stimulant properties. Most alkaloids are toxic, such as pyrrolizidine alkaloids (PAs) help in defense against microbial infection and herbivory. For example, monocrotaline obtained from *Crotalaria*, pyrrolizidine from *Panax quinquefolius*, and senecionine from *Senecio jacobaea* is used as antiherbivore (Irmer et al., 2015; Graser and Hartmann 2000; Stella et al., 2018). Non-protein amino acids, however, do not incorporate into proteins; besides, they move freely, and acts as a protective defense in plants such as mimosine, citrulline, pipecolic acid, and canavanine obtained from *Mimosa*, *Cucumis*, *Calliandra*, and legumes respectively against various kind of stresses such as herbivory and pathogen (Yokota et al., 2002; Moulin et al., 2006; Mazis et al., 2011; Emendack et al., 2018). Cyanogenic glucosides are part of glycosides that break down to produce volatile poisonous substances like HCN that deter feeding by insects and other herbivores. Various plant families possess cyanogenic glucosides *viz.*, Gramineae, Rosaceae, and Leguminosae. For example, amygdalin, dhurrin, linamarin, and lotaustralin extracted from *Prunus* sp, *Sorghum*, and *Lotus japonicum* has been premeditated for their role against drought and as antiherbivore (Mazid et al., 2011; Lai et al., 2015; Emendack et al., 2018; Yadav et al., 2022).

Sulfur-containing secondary metabolites are trivial groups of PSMs that include around 200 compounds. Sulfur-containing PSMs include glucosinolates (GSL), glutathione, glycosphingolipid, phytoalexins, allinin, thionins, and defensins (Venditti and Bianco, 2020). They are directly linked with the defense against microbial pathogens. They are known for their diverse biochemical structures and means of action, which have been reported to give plants a versatile array of chemical defenses to counter various potential enemies (Khare et al., 2020). Glucosinolates are N and S-containing glucosides that are active against unfavorable predators, competitors, and parasites (Bloem et al., 2007). Glucoraphanin, a glucosinolate from *Brassica oleracea*, is used majorly against UV stress (Coleto et al., 2017). Phytoalexins have antimicrobial activities that activate the defense system against these pathogens (Ziegler and Facchini, 2008). For example, brassinin, wasalexins, and camalexin obtained from higher plants show defense against pests and pathogens (Thomma et al., 2002; Abdalla and Mühling, 2019; Rodriguez-Salazar et al., 2017).

PSMs production is, however, not only limited to biotic stress rather an extensive production of PSMs have also been observed during abiotic stress. Drought, flood, and extreme temperature along with heavy metals, and chemicals have a much serious impact on plants (Jha et al., 2019). *Solanum lycopersicum* has been reported to show high activity of phytol (a diterpenoid) upon heat stress (Spicher et al., 2017). Further, other unfavorable condition like cold, is known to slow down the major chemical reactions in plants and under severe circumstances often lead to the death of plants. Pinoresinol, a lignin helps in alleviation during cold stress in almost all land plants (Griffith and Yaish, 2004). Similarly, drought or extreme salinity has reported drastic effects on both physiological and chemical processes (Aftab, 2019, Jan et al., 2021b). Thus, a huge literature supports the effect of abiotic stress on secondary metabolite production.

Since ancient times, the collection of secondary metabolites synthesized from diverse plants has been employed as medicinal ingredients to treat human illnesses. Certain plant species have been discovered to contain a range of active metabolites that participate in the treatment of multiple chronic human diseases, notably cancer, cardiovascular issues, and others. Because they possess antibacterial, antifeedant, and parasiticidal capabilities, as well as being less harmful and inexpensive*. Hypericum perforatum* is employed for its anti-depressant, anti-cancer, anti-inflammatory, anti-viral, and anti-bacterial qualities. This plant exhibits antidepressant drugs including fluoxetine and sertraline as well as other metabolites such as flavonoids, hyperforin, hypericin, and xanthones that increase its medical potential (Shakya, 2016). The *Ipomoea batata L*. is a popular food all over the world. Because it contains so many vitamins and phytochemicals, it has several positive impacts on human health. These phytochemicals have anti-cancer, anti-diabetic, anti-inflammatory, and antioxidant properties. Additionally, beta-carotene, a precursor of vitamin A that aids in the treatment of night blindness and vitamin A insufficiency, is present in sweet potatoes (Ghasemzadeh et al., 2016). There are several secondary metabolites in *Caparis spinosa* that contribute to enhancing biomarkers for diabetes and cardiovascular disease (Zhang and Ma, 2018). The use of secondary metabolites in contemporary medicine may open up new avenues for research into the identification and isolation of the desired pharmacologically active lead chemical in the drug discovery process.

## 3 Transcription factors regulating the biosynthesis of PSMs

Transcription factors (TFs) are class of proteins majorly responsible for regulating gene expression by binding to specific cis-acting elements in the promoter region of target genes. Generally, it has two domains, a DNA binding domain (DBD) and transcriptional activation or repression domain, known to regulate varied cellular processes. mRNA, which comprises 6% of the total genome, is considered the central point of control. In *Arabidopsis thaliana*, over 1600 TFs have been isolated and studied in detail (Gong et al., 2004). Based on the DBD, transcription factors have been named WRKY, bHLH, MYC, MYB, NAC, AP2/ERF, bZIP, and others.

**Figure 3:**
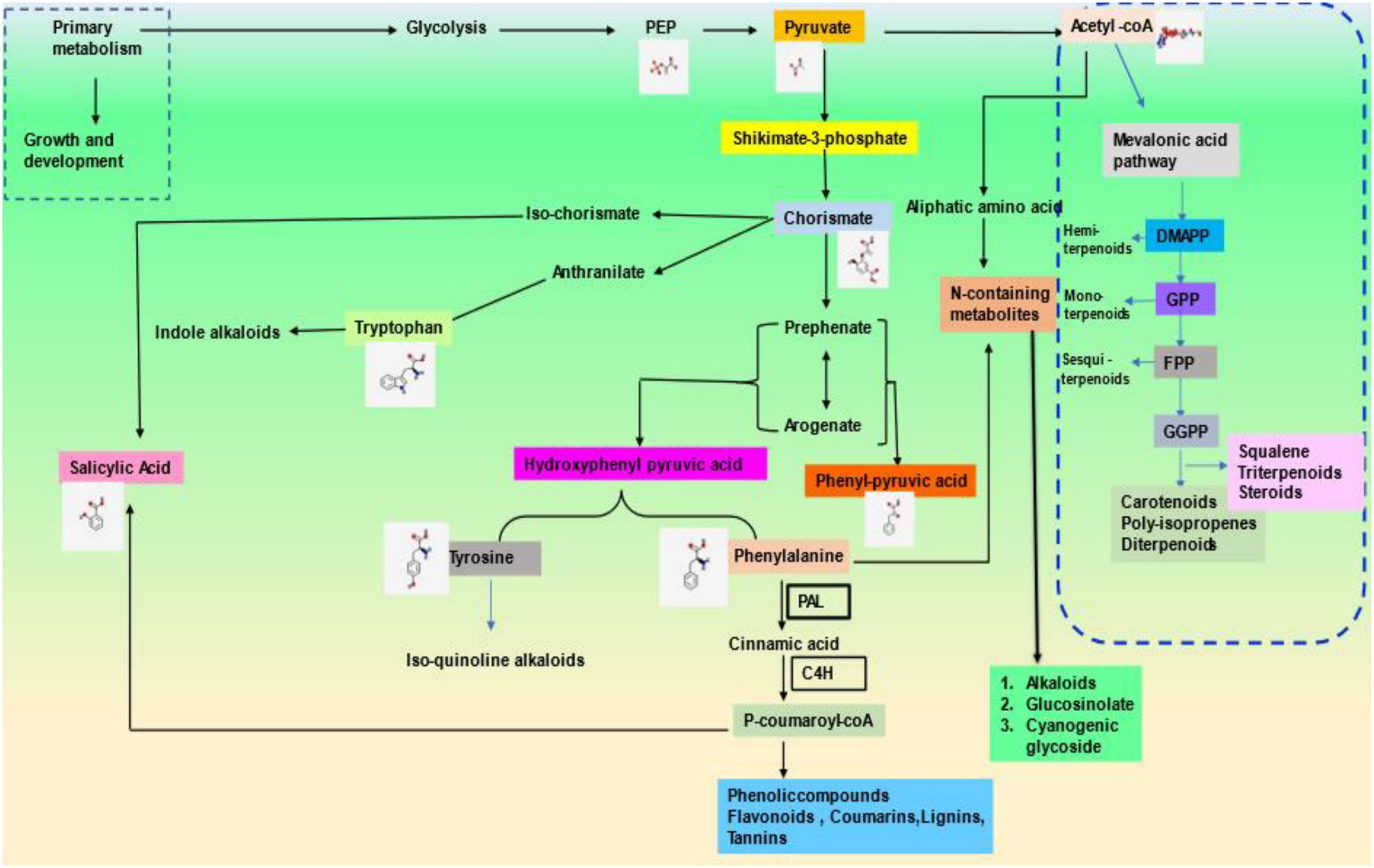
An abundance of various Plants TFs in important economic crops.

TFs have been categorized into various families and superfamilies, which seem to have a distinct role in regulating the stress response, i.e., both abiotic and biotic, a few of which are unique in plants. According to the information on specialized databases such as the Transcription factor database (TFDB), the plants contain more than 2000 genes encoding TFs in the genome. It has been reported in the literature that there are more than 63 families of TFs and about 22 regulators present (Pérez-Rodríguez et al., 2010). Out of these, there are majorly nine Transcription factor families reported recently which have a significant regulatory role in stress tolerance in plants (Chacón-Cerdas et al., 2020) which are identified as ARF, AP2/ERF-ERF, AP2/ERF-DREB, bZIP, BELL, TCP, NAC, WRYK, and ZFP (**Table 1**; **Figure 3**).

**Table 1:** Number of Genes encoding for Transcription factors involved in plant defense (Summarized from Transcription Factor Database, 2022).

S.No.	Plant Species	Transcription Factors							
		WRKY	MYB	AP2	ERF	NAC	bZIP	BHLH	ZF-HD
1	*Amaranthus hypochondriacus*	54	69	15	95	42	56	120	10
2	*Arabidopsis thaliana*	90	168	30	139	138	127	225	18
3	*Artemisia annua*	36	39	4	48	28	35	45	4
4	*Brassica napus*	285	489	57	449	411	264	553	62
5	*Brassica oleracea*	191	306	43	261	271	217	393	47
6	*Brassica rapa*	180	293	42	267	256	200	371	35
7	*Cajanus cajan*	97	179	25	148	96	69	174	20
8	*Catharanthus roseus*	103	110	32	111	121	125	218	15
9	*Cicer arietinum*	94	166	35	144	96	123	197	18
10	*Glycine max*	296	430	99	338	269	352	548	58
11	*Hordeum vulgare*	126	99	34	112	150	156	266	13
12	*Malus domestica*	139	238	45	216	253	114	250	28
13	*Medicago truncatula*	140	185	37	197	123	124	259	18
14	*Nicotiana benthamiana*	133	203	53	266	228	159	277	34
15	*Nicotiana tabacum*	210	319	93	337	280	210	435	46
16	*Oryza sativa*	109	121	27	138	158	94	169	15
17	*Phaseolus vulgaris*	102	181	31	155	106	105	203	19
18	*Solanum lycopersicum*	81	140	27	137	101	70	161	22
19	*Solanum melongena*	65	105	23	132	95	57	121	15
20	*Solanum tuberosum*	125	126	45	185	131	95	206	19
21	*Sorghum bicolor*	134	145	42	172	180	166	297	21
22	*Triticum aestivum*	171	263	43	181	263	186	324	20
23	*Vigna angularis*	92	120	27	253	115	122	192	30
24	*Vigna radiata*	88	117	21	113	82	63	153	15
25	*Vigna unguiculata*	22	26	5	38	20	34	61	6
26	*Zea mays*	161	203	54	204	189	216	308	26

This review focuses on the role of six TFs, namely, WRKY, NAC, AP2/ERF, MYB, bZIP, and bHLH, that are majorly intricated in regulating and synthesizing secondary metabolites under biotic stress in economically important crops. A few reviews have suggested the diverse role of TFs in regulating plant defense, but there has been a meager understanding of the role of TFs in biosynthesis and regulation of PSMs under biotic stress and how miRNAs regulate them. Therefore, this review has been planned to focus on transcription factors involved in PSMs biosynthesis during plant defense and their regulation by miRNAs in both model plants and economically important crops.

### 3.1 WRKY TF

WRKY is the foremost characterized class of plant TF, which regulates various plant processes related to development, physiology, metabolism and plant defense (Chen et al., 2017). From the discovery of WRKY in *Ipomea batata* in the 1990s to the diversified presence in many of the eukaryotic organisms, be it fungi, amoebae, or higher eukaryotic plants (Ülker and Somssich, 2004; Yamasaki et al., 2005; Zhang and Wang, 2005; Rushton et al., 2010). The genome-wide characterization and analysis in model plants stipulate a widely authenticated and accepted classification system of the WRKY TF family specific to plants. It consists of 60 amino acids in their highly conserved DBD (called WRKY domain) (Eulgem et al., 2000). The WRKY domain comprises the WRKYGQK motif at N- terminus, a highly conserved motif, and a zinc-finger region at C -terminus binding to W-box (TTG ACT/C), which shows higher affinity towards DNA binding, although other binding sites also have been reported (Ciolkowski et al., 2008; van Verk et al., 2008; Li et al., 2010; Schmutz et al., 2010; Ishiguro et al., 2011; Brand et al., 2013; Rinerson et al., 2015; Cia et al., 2018). The number of genes encoding for the WRKY family varies amongst plants being 171 WRKY genes in wheat,161 in maize, 94 in chickpea, 171 in rice, etc (**Table 1**).

WRKY TFs have a diverse role in various plant processes, such as development, germination, seed dormancy and were first reviewed in 2010 (Rushton et al., 2010). Since then, its role has been thoroughly studied and experimented with. However, its significance in regulating stress response is of prime importance. It has been known to regulate and interconnect multiple environmental stimulations (Rushton et al., 2010; Rushton et al., 2012; Schluttenhofer and Yuan, 2015; Banerjee and Roy choudhury, 2015; Phukan et al., 2016). There has been a detailed review of WRKY TFs in various abiotic stresses such as cold (Zou et al., 2010; Guo et al., 2015; Dai et al., 2016; Yu et al., 2016), drought (Tripathi et al., 2014; He et al., 2016; Liu J et al., 2016; Chu et al., 2015; Li R et al., 2015; Raineri et al., 2015; Yan et al., 2015; Wang Y et al., 2016; Wani et al., 2018; Jha et al., 2019), salt (Wang L et al., 2014; Ding et al., 2015; Ma et al., 2015; Bai et al., 2018), osmotic stress (Mao et al., 2011; Li H et al., 2013; Marchive et al., 2013; Phukan et al., 2016; Jiang et al., 2017; Li et al., 2020), heat (Grover et al., 2013; Cai et al., 2015; He et al., 2016; Wang Y et al., 2016; Ohama et al., 2017; Sita et al., 2017; Cheng Z et al., 2021) and many more in economically important crops.

Many previous studies have described that plants harbour evolutionarily conserved defense mechanisms against pests (Peng et al., 2011; Sun et al., 2015; Kloth et al., 2016; Yang et al., 2017; Jha et al., 2020) and pathogens such as fungus (Turck et al., 2004; Guo et al., 2011; Inoue et al., 2013; Dang et al., 2014; Zhang, 2014), and bacteria (Deslandes et al., 2002; Kim et al., 2008) (**Table 2**). Most WRKY TF have been known to show negative regulation towards stress, while only a few exhibit positive responses during stress (Kim et al., 2008; Xing et al., 2008).

**Table 2:** Role of Transcription factors in the biosynthesis of plant secondary metabolites in plants.

S.No.	Biotic stress	Families	Transcriptional Factors	Plant Species	Secondary Metabolites	References
1.	Pathogen Attack	AP2/ERF	PnERF1	*P. notoginseng*	Saponins	Deng et al., 2017
		WRKY	StWRKY1	*S. tuberosum*	HCAAs	Yogendra et al., 2015
		bHLH	TSAR1/TSAR2	*M. falcata*	Saponins	Mertens et al., 2016
		MYB	CsMYBF1	*C. sinensis*	Flavonoids and HCAAs	Wang et al., 2018
		MYB	AtMYB11/12/111	*A. thaliana*	Flavonoids	Mishra et al., 2010
		WRKY	WsWRKY1	*W. somnifera*	Phytosterol	Singh et al., 2017
		WRKY	TaWRKY70	*T. aestivum*	HCAAs	Kage et al., 2017
		WRKY	SsWRKY18/40	*S. sclarea*	Diterpenoids	Alfieri et al., 2018
		MYB	OsMYB30/55/110	*O. sativa*	HCAAs	Kishi-Kaboshi et al., 2018
		MYB	AtMYB11/12/111	*A. thaliana*	Flavonoids	Mishra et al., 2010
		bZIP	OsTGAP1	*O. sativa*	Diterpenoid phytoalexins	Miyamoto et al., 2015
		bZIP	OsbZIP79	*O. sativa*	Diterpenoid phytoalexins	Miyamoto et al., 2015
		bZIP	SlHY5	*S. lycopersicum*	Anthocyanin Monoterpenoids	Liu et al., 2018
		bHLH	DPF	*O. sativa*	Diterpenoid phytoalexins	Yamamura et al., 2018
		MYB	AtMYB12	*A. thaliana*	Flavonoids	Hamamouch et al., 2020
2.	Nematode Attack	WRKY	GmWRKY136, GmWRKY53, GmWRKY86	*G. max*	SA	Yang et al., 2017
		WRKY	SlWRKY45, SlWRKY3, SIWRKY70	*S. lycopersicum*	Phenolics	(Chinnapandi et al., 2017; Chinnapandi et al., 2019
		MYB	AtMYB75	*A. thaliana*	SA	Shen X et al., 2018
		MYB	TaMYB19, TaMYB2, TaMYB44	*T. aestivum*	SA	Shen X et al., 2018
3.	Insect Attack Toxic against herbivory	WRKY	OsWRKY45, OsWRKY46	*O. sativa*	Ethylene, H_2_O_2_	Huang et al., 2016
		AP2/ERF	NtERF32	*N. tabaccum*	Nicotine	Sears et al., 2014
		AP2/ERF	EREB58	*Z. mays*	Sesquiterpenes	Li M et al., 2015

WRKY TFs have been reported to regulate the biosynthesis of several secondary metabolites like phenols, lignin, flavonoids, tannins *etc.*, and their inducible expression analysis revealed a role in regulating defense-related PSM biogenesis (Guillaumie et al., 2010; Wang et al., 2010; Grunewald et al., 2012; Phukan et al., 2016). For instance, WRKY3 and WRKY6 have been reported for their role in the biosynthesis of volatile terpenes in tobacco (Skibbe et al., 2008). Artemisinin, an isolated sesquiterpenoid lactone majorly used as an antimalarial drug, is extracted from *Artemisia annua* (Meshnick et al., 1996); it has shown upregulation in the presence of WRKY1 (Asadollahi et al., 2008). Similarly, AaWRKY17 is reported to positively regulate artemisinin synthesis in providing resistance against *Pseudomonas syringae* (Chen et al., 2021).

Another PSM, Hydroxycinnamic acid amide (HCAA), is a phenol derived from the phenylpropanoid pathway, which is majorly involved in lignin biosynthesis and is derived from phenylalanine (Humphreys et al., 1999; Vogt, 2010; Rio et al., 2021). WRKY1 in late blight-infected potato plants binds to its promoter sites (Yogendra et al., 2015). Similarly, associated genes like ACT, DGK, and GL1 are activated in wheat by TaWRKY70, upon encountering fungi (Kage et al., 2017). Red rot caused by *Fusarium* in barley, HvWRKY23 promotes the expression of genes like CHS, HCT and PAL, which further induces HCAA biosynthesis (Karre et al., 2019). Likewise, StWRKY8 enhances the expression level of COR2, NCS, and TyDC genes majorly involved in the production of benzyl iso-quinoline alkaloids in potatoes; against bacterial attack (Yogendra et al., 2017). In addition, SsWRKY18, SsWRKY40, and SsMYC2 play a regulatory role in the production of abietane-type diterpenes in *Salvia sclarea*, which exhibits antimicrobial (Alfieri et al., 2018).

Phytoalexins, another secondary metabolite, belonging to the stilbene family, are considered significant regulators of plant defense (Jiang et al., 2010; Ahuja et al., 2011). Resveratrol in grapes has been the first reported class of phytoalexins (Lanz et al., 1991). VvWRKY8 has been found to modulate the biosynthesis of resveratrol in grapes which ultimately can be engineered to enhance resistivity (Jeandet et al., 2019). It was shown to negatively regulate the stilbene synthase gene (Jiang et al., 2019). ZmWRKY79 in maize caused increased phytoalexin accumulation under biotic stress conditions (Fu et al., 2017). Similarly, GaWRKY is responsible for the increased production of gossypol, which shows anti-feeding properties in cotton (Xu et al., 2004). WsWRKY1, in *Withania*, has been reported to significantly reduce phytosterol accumulation, resulting in decreased resistance to bacteria, fungi, and insects (Singh et al., 2017). In addition, SsWRKY18, SsWRKY40, and SsMYC2 show regulation in accumulating abietane (diterpene) in *Salvia sclarea*, which exhibits antimicrobial properties (Alfieri et al., 2018). Another example CjWRKY1 from *Coptis japonica* controls berberine biosynthesis (Kato-Noguchi and Takeshita, 2013).The regulatory roles of WRKY TFs production have been explored extensively. This information can be employed to engineer biotic stress resistance in transgenics.

### 3.2 MYB

MYB (Myeloblastosis) TFs are essential and functionally diverse proteins in eukaryotes (Jia et al., 2004). It is widely studied in plants, highlighting its importance in the system. Scientific investigations on *Zea mays* led to the isolation of the first MYB-TF in 1980s, which was homologous to the MYB proto-oncogene present in animals (Paz-Ares et al., 1987). MYB proteins typically have two distinct regions: N-terminus with a conserved DNA binding MYB domain, and a variable C-terminus making these TFs functionally diverse (Frampton et al., 1991). MYB domains generally contain 1–4 imperfect repeats of 50–53 amino acid residues each. Each repeat contains three α-helices, of which the second and third helices are responsible for specific gene recognition (Ogata et al., 1996). The third helix is the central part that makes contact with the major groove of DNA (Jia et al., 2004). Based on the number of repeats and their position, MYB TFs are categorized into four classes:1R (R1/2, R3-MYB), 2R (R2R3-MYB), 3R (R1R2R3-MYB), and 4R (R1/R2-like repeats) (Dubos et al., 2010).

Various growth and developmental processes in plants involve MYB TFs, such as flower color development in *Glycine max* (Takahashi et al., 2013) and signal transduction in *A. thaliana*, *Zea mays*, *Oryza sativa*, and cassava (Bakhshi and Arakawa, 2006; Raffaele et al., 2006; Liao et al., 2016). MYB TFs also control cell cycle and morphogenesis in various plants (Joaquin and Watson, 2003; Cominelli and Tonelli, 2009; Baumann et al., 2007). In addition to these diverse roles, MYB has also been well documented to regulate secondary metabolite synthesis in many plants, for instance, *Camellia sinensis, A. thaliana*, and *Medicago truncatula* (Verdier et al., 2012; Ambawat et al., 2013; Patra et al., 2013; Liu et al., 2015; Nguyen et al., 2016; Zhao et al., 2022). This production and regulation of secondary metabolites help combat enemies during biotic stress. Whenever any pathogen or insect attacks a plant, it tends to fortify its cell walls and membranes to halt the invasion (**Table 2**) (Kim et al., 2003; Kim et al., 2013). Several MYB factors are shown that control lignin biosynthesis (Zhu et al., 2020; Xiao et al., 2021). CmMYB15 in *Chrysanthemum morifolium* binds to AC elements of lipid synthesis gene promoter to regulate lignin biosynthesis during aphid invasion. Overexpressing CmMYB15 led to a reduction in aphid proliferation (An et al., 2019). MdMYB30, a His MYB-TF was found to improve disease resistance by regulating wax biosynthesis in apples (Zhang et al., 2019). In some plants, secondary metabolites like phytoalexins are produced to inhibit the growth of pathogens. Sorghum MYB TF y1 (yellow seed 1) protects maize from *Colletotrichum sublineolum infection* by synthesizing 3-deoxyanthocyanidin phytoalexins (Ibraheem et al., 2015; Chen et al., 2018). VdMYB1, a member of the R2R3-MYB TF, induce a defense response in the grapevine against *Erysiphe necator* fungal infection by activating stilbene synthase 2 gene (VdSTS2) (Yu et al., 2019).

MYB TFs are also reported to modulate the production of essential PSMs such as flavonoids, glycolipids, hydroxycinnamic acid amides (HCAAs), and proanthocyanidins during biotic stress. AtMYB29 and AtMYB76 are correlated to the synthesis of aliphatic glucosinolate in *Arabidopsis*. On the other hand, AtMYB34, AtMYB51, and AtMYB122 are responsible for indole glucosinolate (IG) accumulation which is related to tryptophan biosynthesis genes (CYP79B2, CYP79B3, and CYP83B1) expression (Dubos et al., 2010). *Arabidopsis* triple mutant MYB-34/MYB-51/MYB122 has shown increased *Plectosphaerella cucumerina* susceptibility due to reduced IG levels (Frerigmann et al., 2016). MYBs are well known to control anthocyanin production. In *Arabidopsis thaliana*, AtMYB113, AtMYB114, AtMYB75, and AtMYB90 modulate phenylpropanoid pathway to alter anthocyanin levels (Gonzalez et al., 2008). Similarly, in Asiatic hybrid lily plants, anthocyanin can be regulated by MYB6 and MYB12 (Yamagishi et al., 2010). In citrus, CsMYBF1 upregulates chalcone synthase (CHS) gene during certain flavonoid production (Liu J et al., 2016). In *Camellia sinensis*, CsMYB2/CsMYB26 have been found to promote flavonoid accumulation by binding to CsF30H and CsLAR gene promoters, thus improving resistivity against *Exobasidium vexans*, the causative agent of blister blight (Nisha et al., 2018). It was reported that *Arabidopsis* AtMYB11, AtMYB12, and AtMYB111 play an essential role in flavonoid production and that heterologous expression of AtMYB TFs can be heterologously expressed to increase flavonoid content in several other plants (Wang Y et al., 2016).

Besides significant positive regulatory roles, AtMYB75 has a negative impact on flavonoid synthesis, which results in reduction of kaempferol-3,7-dirhamnoside. This reduction makes the plants susceptible to insect attack (Onkokesung et al., 2014). In rice, phenylpropanoid pathway genes are upregulated by MYB30, MYB55 and MYB110, which accounts for increased HCCAs content (Kishi-Kaboshi et al., 2018). In comparison, the proanthocyanidin biosynthesis genes are downregulated by VvMYBC2-L1 in grapes, negatively regulating proanthocyanidin biosynthesis (Huang et al., 2014). Moreover, enhanced proanthocyanidins can elevate resistance against wounding in *Rosa rugosa* by the action of RrMYB5 and RrMYB10 (Shen Y et al., 2019). In Poplar trees, PtMYB115 helps in enhancing resistance during *Dothiorella gregaria* infection by binding to ANR1 & LAR3 genes to elevate proanthocyanidin levels (Wang et al., 2017). Given the ample information in this field, it is worth discussing how MYB TFs regulate these PSMs’ production, which would help to generate generate future stress-tolerant crops of economic value (Ambawat et al., 2015; Dubos et al., 2010).

### 3.3 AP2/ERF

AP2/ERFs (APETALA2/Ethylene Response Factor) consist of an AP2 DNA binding domain consisting of 40–70 conserved amino acids, which was first discovered in floral homeotic gene APETALA2 (AP2) of *A. thaliana* (Feng et al., 2005; Nakano et al., 2014). It is categorized into four subfamilies based on the variation of conserved domains: AP2, DREB, RAV, and ERF. Reportedly AP2 comprises two AP2 domains, DREB contains one AP2 and an A-subfamily, RAV consists of one AP2 and a B3 domain, while ERF has one AP2 and a B-subfamily (Zhou et al., 2016). In addition to imposing transcriptional and post-translational control upon growth, developmental, hormonal, and stress-responsive genes, AP2/ERFs transcription factors also govern biosynthesis and regulation of secondary metabolites (Mizoi et al., 2012; Licausi et al., 2013; Gibbs et al., 2015; Chandler, 2018, Xie et al., 2019).

Scientists have also reported the role of ERF TFs in plant defense against different pathogens, which can be employed to enhance pathogen resistance in crops by engineering pathways regulating specialized secondary metabolites. In *Catharanthus roseus*, ORCA1 and ORCA2 are two AP2/ERF proteins that bind to the promoters of biosynthetic genes of terpenoid indole alkaloid (TIA) (Menke et al., 1999). JA-induced ORCA3-TF binding to the JERE element of the promoter then induces two TIA biosynthetic genes encoding tryptophan decarboxylase and strictosidine synthase (vander Fits and Memelink, 2000). ORCA3, 4, and 5 in *C. roseus* interact with each other and act on MAPK to regulate TIA biogenesis (Paul et al., 2017; Paul et al., 2020). These TIAs, like catharantins, are involved in fungal and insect resistance by protecting the leaf surface of *C. roseus* from pathogenic infections and insect infestation (Roepke et al., 2010).

Steroidal glycoalkaloids (SGAs) are cytotoxic, which benefits plants to resist pathogen and insect invasion (Friedman, 2002; Friedman, 2006; Itkin et al., 2013; Nakayasu et al., 2017). In tobacco and *C. roseus*, GLYCOALKALOID METABOLISM 9 (GAME9) is found to regulate the biosynthesis of SGAs. Modifications in gene expression for SGA production and mevalonate pathway due to overexpression and knockdown, respectively. (Cárdenas et al., 2016). GAME9/JRE4 bind to the promoter region of SGA biosynthetic gene, which results in the upregulation of GAME, CAS, HMGR, SGT, and C5-SD genes (Thagun et al., 2016). GAME9 is reported to be a regulator of SGA biosynthesis to provide *Spodoptera litura* resistance in tomatoes (Nakayasu et al., 2018). Similar SGAs accumulation and regulation were discussed in tomatoes and potatoes (Cárdenas et al., 2016; Thagun et al., 2016; Nakayasu et al., 2018).

Nicotine, a tobacco alkaloid, is a repellent that facilitates plant protection against herbivores (Steppuhn et al., 2004). The AP2/ERF gene has been reported to be responsible for nicotine biosynthesis in tabacco. Accumulating nicotine biosynthesis results in positive action of NtERF189 and ORC1 on the associated genes, and NtMYC2, a JA inducer, also has the same effect (Shoji and Hashimoto, 2011; De Boer et al., 2011). Expression of putrescine N-methyltransferase coding gene, NtPMT1a, involved in nicotine biosynthesis, was found to be downregulated by NtERF32 (Sears et al., 2014). ERF located at NIC-2 locus regulates biosynthesis in tobacco of nicotine against herbivory (Shoji et al., 2010 and De Boer et al., 2011), NtORC1/ERF221 and NtJAP1/ERF10 in tobacco have been positively regulated by PMT gene which synthesizes nicotine and pyridine alkaloids (De Sutter et al., 2005; Thagun et al., 2016).

Taxol, an anticancer and plant defense compound, provides resistance against *Phytophthora capsici* infection (Young et al., 1992). TcERF12 and TcERF15, are regulators of taxol biosynthesis, the former having adverse effects while the latter having positive action (Zhang et al., 2015). Another class of antimicrobial metabolites is the hydroxycinnamic acid amides (HCAAs), which confer resistance to many fungi like *Alternaria brassicicola* and *Botrytis cinerea* on plants (Meraj et al., 2020). ORA59, another AP2/ERF TF, governs HCAA production by controlling AtACT (agmatine coumaryl transferase) (Li et al., 2018).

Saponins have been shown to have a variety of functions, including serving as phytoprotectants (Papadopoulou et al., 1999; Avato et al., 2006). In *Panax notoginseng*, PnERF1 was discovered to bind to the saponin biosynthesis gene (HMGR, FPS, DS, SS) promoters that increase total saponin content in overexpressing lines (Deng et al., 2017). Furthermore, lignin is an essential defensive substance as a fundamental component of the plant cell wall. GbERF1 prevents V*erticillium dahliae* infection in *Gossypium barbadense* by increasing lignin production (Guo et al., 2016). Genome-wide & transcriptome analysis confirmed that gypenosides (a triterpenoid saponin) in *Gynostemma* are regulated under biotic stress (Xu et al., 2020). Similarly, in *Solanum melongena*, Smechr0902114, and Smechr1102075, which belong to ERF TF, were reported to regulate anthocyanin biosynthesis (Li et al., 2021).

Several ERFs have been known to activate the transcription of various disease-responsive genes such as PR (pathogenesis-related), glucanase, chitinase, etc. They have also been reported in the regulation of abiotic stress (Yi et al., 2004; Seo et al., 2010; Fukao and Mito et al., 2011; Fahad et al., 2017; Li Z et al., 2015; Verma et al., 2016; Chen et al., 2017; Nolan et al., 2017; Van den Broeck et al., 2017; Ye et al., 2017; Bechtold and Field, 2018; Chen et al., 2021) and biotic stress (Lorenzo et al., 2003; Zarei et al., 2011; Moffat et al., 2012, Zhang et al., 2009; Liu et al., 2011; Liu et al., 2012; Müller and Munné-Bosch, 2015; Abiri et al., 2017). Many elicitors (Dzhavakhiya and Shcherbakova, 2007) originating from yeast, fungi, and bacteria have been lately studied to induce the synthesis of PSMs (Dzhavakhiya et al., 2007; Hu et al., 2003; Sang et al., 2010; Almagro et al., 2012; Awad et al., 2014; Naik and Al-Khayri, 2016; Jan et al., 2021a).

### 3.4 NAC

NAC [NAM (no apical meristem)], ATAF1/2, and CUC2 (cup-shaped cotyledon) are TF families that are quite connected to plant stress response (Ahuja et al., 2010). NAC proteins consist of a highly diverse C-terminal transcription regulatory (TR) domain, a conserved N-terminal binding domain, and a transmembrane domain (Puranik et al., 2012; Tweneboah and Oh, 2017). Functional genomics studies have revealed that NAC genes are well distributed in different plant species. According to the latest Transcription Factor Database (TFDB) records, 138 non-redundant NAC genes exist in the model plant, i.e., *A. thaliana*, around 263 in *Triticum aestivum*, 158 in *Oryza sativa* and 189 in *Zea mays* and many others (**Table 1**).

The NAC gene families show extensive regulation under environmental stresses (Nakashima et al., 2012; Nuruzzaman et al., 2013). Many investigations on these lines revealed the role of NAC TFs during abiotic stress; however, in recent years, biotic stress has also gained attention among plant scientists. It has also been reported that NAC regulates defense responses against microbe and insect attacks (Puranik et al., 2012). During pathogen attack, NAC induces hypersensitive response in essential crops such as *Glycine max* (Faria et al., 2011), sorghum (Zhang et al., 2013), wheat (Saad et al., 2013), barley (Chen et al., 2013), rice (Liu et al., 2018) and other plants (Zhou et al., 2018). PSMs production during stress is one of the prime defense mechanisms controlled by NAC TFs. Among them, phytoalexins are low molecular mass PSMs produced during pathogen attacks (Ahuja et al., 2012). In *A. thaliana*, ANAC042 confers plant resistivity to *Alternaria brassicicola* attack by binding to the promoter of camalexin biosynthetic genes (Saga et al., 2012; Duan et al., 2017). Camalexin (3’-thiazol-2’-yl-indole) is a well-known primary phytoalexin released after microbial infection in many plants (Nguyen et al., 2022). Glyceollin, another isoflavonoid-derived phytoalexin from soybean is positively regulated by GmNAC42-1 which imparts resistivity to plants (Jahan et al., 2019).

NAC TFs also regulate ROS homeostasis during stress conditions by regulating certain PSMs. The Arginine decarboxylase gene (ADC) controls putrescine biosynthesis, which can regulate ROS homeostasis. In *Poncirus trifoliata*, PtrNAC72 was found to regulate the expression of ADC (Wu et al., 2016). Furthermore, glutathione biosynthesis is regulated by MfNAC in *Medicago falcate*, which controls the expression of glyoxalase-1 (GLO1) gene (Duan et al., 2017).

In *Hevea brasiliensis*, latex biosynthesis is governed by HbNAC1, which shows binding affinity towards the CACG motif present in the promoter region of small rubber protein. This may have a potential role in healing wounds caused by herbivores (Cao et al., 2017). Many NAC TFs have also been found to regulate specific genes involved in defense systems negatively. From Norway spruce (*Picea abies*), PaNAC03 has been reported to regulate flavonoid biosynthesis genes like CHS, LAR3 negatively, and F3’H, consequently providing resistance to *Heterobasidion annosum* (Danielsson et al., 2011). Similarly, ANACO32 negatively regulates anthocyanin biosynthesis genes like DFR, LODX, and ANS (Mahmood et al., 2016). Flavonoids and anthocyanin have been well-documented as important secondary metabolites produced during the plant-pathogen arms race (Shah and Smith, 2020, Sivankalyani et al., 2016).

### 3.5 bZIP

The basic-leucine-zipper (bZIP) proteins, a class of TFs, are crucial regulators of plant growth and development. It consists of a conserved bZIP domain composed of contiguous α-helix. It consists of a primary region (18 amino acids), NLS, and sequence-specific DNA binding region at the N-terminus, which is highly conserved, followed by leucine zipper (heptad repeats of hydrophobic amino acids) at the C-terminus, which is less conserved (Jakoby et al., 2002; Nijhawan et al., 2008; Landschulz et al., 1988; Ellenberger et al., 1992; Furihata et al., 2006; Liao et al., 2008). The bZIP members are identified in many species, such as rice (94), wheat (186), maize (216), Arabidopsis (127), and so on (**Table 1**; **Figure 3**).

bZIP TFs are widely associated with plant stresses (Alves et al., 2013; Li et al., 2017). However, its function has been extensively studied in regulating abiotic stress (Banerjee and Roy choudhury, 2017; Baillo et al., 2019) such as drought, chilling, heat, osmosis, high salinity, etc., (Golldack et al., 2014). They are more diverse in plants as compared to microorganisms and animals (Maillet et al., 2021). The role of bZIP under biotic stress such as herbivory, wounding, pathogen attack, etc., and the plant’s protection *via* phytohormone signaling pathways or hypersensitive responsive defense mechanisms have been remotely studied (Zhang H et al., 2017; Joo et al., 2019). The molecular regulation of bZIP during a pathogen attack has been very well reviewed by Alves et al., 2013. Similarly, the regulatory roles of bZIP against resistance to pathogens have already been reported (Noman et al., 2017). For example, four bZIP genes have been reported in *Glycine max* (GmbZIPE1, GmbZIPE2, GmbZIP105, and GmbZIP62) that defend plants against Asian soybean rust (ASR), which in turn produces PSMs (Alves et al., 2015). In cassava, it has been revealed that *Xanthomonas axonopodis pv. manihotis* induces MebZIP3 and MebZIP5 upon infection (Li et al., 2017).

Okada et al., 2009 have shown that OsTGAP1 in rice binds to the promoter *of OsKSL4* and *OsCPS4* and enhances terpenoid phytoalexins accumulation, improving resistivity against blast disease. Some specific bZIP proteins are also involved in pharmaceutically important SMs. SmbZIP20 and SmbZIP7 are known to regulate tanshinone in *Salvia miltiorrhiza* (Cao et al., 2018). Similarly, AabZIP1 regulates artemisinin synthesis in *Artemisia annua* (Shen Q et al., 2019). Li et al., 2017 have engineered cassava bacterial blight-resistant plants by overexpressing MebZIP3 and MebZIP5. There are many reports explaining the role of bZIP in plant defense against pathogens, but their importance during plant defense against insects and nematode attacks is still less explored; these areas have great potential for further exploration.

### 3.6 bHLH TFs

bHLH (basic-helix-loop-helix) TFs are one of the TF families found in eukaryotes, mainly plants (Carretero-Paulet et al., 2010). It consists of two connected regions the N-terminal region (basic in nature) followed by the HLH domain (40-50 amino acid residues) and a highly conserved HER motif (Atchley et al., 1999; Massari and Murre, 2000; Toledo-Ortiz et al., 2003) that binds to DNA and regulates transcription both positively and negatively (Yang et al., 2020). It was first reported in *Zea mays* for regulating abiotic stress response (Ludwig et al., 1989).

Many studies have deciphered the role of bHLH concerning environmental stresses (Babitha et al., 2015; Jin et al., 2016; Qian et al., 2021). However, reports of the role of bHLH in biotic stress are scarce (Meraj et al., 2020). bHLH has been known to regulate hormonal signaling pathways, especially Jasmonic acid that triggers plant immunity (Tian et al., 2015). They have also been found to regulate stress responses by controlling the production of secondary metabolites like flavonoids, anthocyanins, glucosinolates (GLs), phytoalexins, etc. (Sun et al., 2018).

Generally, bHLH interacts with MYB TFs to control specific gene expression. It has been explicitly reported in *Arabidopsis*, bHLH-04 to 06 interaction with MYB51 regulates the biosynthesis of GLs and the biosynthesis of anthocyanin and flavonoids through the phenylpropanoid pathway (Frerigmann et al., 2014). Similarly, GLABRA3 (GL3), Enhancer of GLABRA3 (EGL3), and Transparent Testa 8 (TT8) proteins of the bHLH family were found to activate anthocyanin biosynthetic genes after binding to MYB transcriptional complex (Dubos et al., 2010). MYC2, another bHLH member, has regulatory roles in jasmonic acid signaling by binding to Jasmonic acid responsive element (JARE) called ORCA-3, which acts as a promoter and enhances alkaloid biosynthesis (Zhang et al., 2011). MdMYC2 in *Arabidopsis* upregulates certain anthocyanin-related genes, promoting sesquiterpene synthesis (Nemesio-Gorriz et al., 2017). Similarly, in rice, diterpenoid phytoalexin factor (DPF) has been seen to regulate the production of diterpenoid phytoalexin, which could have a potential role during pathogen attack (Yamamura et al., 2015).

In *Nicotiana benthamiana*, NbbHLH1, NbbHLH2, and NbbHLH3 have been implicated in nicotine biosynthesis through virus-induced gene silencing (VIGS) by binding to the promoter of putrescine N-methyltransferase (Todd et al., 2010). HMGR activation in *Medicago truncatula* is accountable for saponin synthesis, which TSAR1 and TSAR2 regulate. (Mertens et al., 2016). Avenacin, an important saponin, has been found to check the fungal growth in plants. As mutants of *Avena strigosa* deficient in avenacin become susceptible to *Gaeumannomyces graminis* var. *tritici* infection (Papadopoulou et al., 1999). An IAA-LEUCINE RESISTANT3 (ILR3) has been reported to have a significant role against nematode infestation by regulating aliphatic GLs production (Samira et al., 2018). Although many stress-related TFs have been reported, the exact molecular mechanism of TFs regulating these PSMs is still a research hotspot.

## 4 MiRNAs - master regulators of plant defense response

As discussed earlier, microRNAs (miRNAs) are a class of small (20-24 nucleotide long), non-coding RNAs that play a presiding part in regulating gene expression post-transcriptionally through degrading their target mRNA or halting translational machinery (D. Baulcombe, 2004). During these years, the miRNA has gained massive attention by scientists due to its widespread role in plant development and stress tolerance (Yang T et al., 2007). Emerging evidences suggest its potential role in regulating several defense-related genes during biotic stress (Ruiz-Ferrer and Voinnet, 2009; Kumar, 2014; Šečić et al., 2021). Typically, miRNAs target specific genes that are directly or indirectly involved in any defense pathway when a plant faces pest and pathogen attack. During stress, many miRNAs have been found to target genes involved in the production of defense-related compounds like secondary metabolites (Owusu Adjei et al., 2021). Meanwhile, miRNAs have also been reported to control many transcription factors that control the production of defense products (Javed et al., 2020). Thus, miRNAs are crucial factor to be emphasized during stress as they also regulate the regulators i.e, TFs.

### 4.1 MiRNAs involved in PSM production

Secondary metabolites are one of the prime plant products used by plants to combat pest and pathogen attacks. Thus, differential regulation of these SMs is quite obvious during stress. miRNAs have been found to chiefly regulate these PSMs (Owusu Adjei et al., 2021). Kettles et al. (2013) have shown that miRNA mediates the regulation of secondary metabolites like camalexin, which provides resistivity to *Arabidopsis* against green peach aphids. miR393 is well documented to be involved in plant defense by shifting resources to the glucosinolate pathway from the camalexin pathway resulting in the production of the most effective PSMs (Robert-Seilaniantz et al., 2011). It has also been found that the flg22 application leads to the surge of miR393, suggesting involvement in defense.

miR169 is known for its participation in anthocyanin biosynthesis in *Piper nigrum* by targeting UGT79B1, an anthocyanidin 3-O-glucoside 2’’-Oxylosyltransferase enzyme; along with 73 other miRNAs indicating a significant role in regulating metabolic pathway during biotic stress (Ding et al., 2021). Additionally, miR828 and miR858 target repressors of MYB, which leads to the activation of anthocyanin biosynthesis pathway and accumulation of anthocyanin and flavonol.VvMYB114 is targeted by miR828 and miR858 that represses various enzymes such as DFR (Dihydroflavonol-4-reductase) and UFGT (UDP-glucose: flavonoid 3-O-glucosyltransferase) in grapes (Tirumalai et al., 2019). Sometimes miRNAs can also target certain genes to regulate PSMs production during biotic stresses. The squamosal promoter-binding protein-like (SPL) 9 gene can be targeted by miRNA156, which contributes to *Arabidopsis*’ stress resistance by promoting the massive accumulation of anthocyanins (Cui et al., 2014).

### 4.2 MiRNAs regulating TFs

TFs play a prime role during any defense response, thus being the preferred target of miRNA to bring changes at the molecular level (Li and Zhang, 2016). Many reports suggest the role of miRNA in controlling various stress-responsive TFs (Lima et al., 2012; Koroban et al., 2016; Samad et al., 2017). Additionally, a sufficient number of miRNAs have been documented to regulate TFs, such as MYB, NAC, WRKY, ERF, ARF, and bHLH, which have been previously discussed to control the production of secondary metabolites. In wheat, miR164 has been found to negatively interact with NAC21/22, which ultimately enhances the susceptibility to stripe rust brought on by the pathogen, *Puccinia striiformis* (Zheng et al., 2016).

Similarly, to target leaf spot fungus, *Alternaria alternate*, Md-miRNA395 and Md-miRNA156ab were reported to target MdWRKY26 and MdWRKYN1, respectively (Zhang Q et al., 2017). Sly-miR1127 is known to regulate slyWRKY75 during *Botrytis cinerea* infection in tomato plants (López-Galiano et al., 2017). *Magnaporthe oryzae* stimulated the expression of miR5819 and miR5075 in rice, and it was discovered that these miRNAs specifically aimed at OsbZIP38 and OsbZIP27, respectively. PvAP2-ERF genes in *Phaseolus vulgaris* L. have also been revealed to be the target of miRNAs from other plant species, with miR156, miR172, and miR838 possibly being involved in the regulation of ERF/AP2 (Kavas et al., 2015). During rhizobial infection, the concentration of miR160 and miR164 that influence ARF (ARF17, ARF10, and ARF16) and NAC respectively changed (Basel Khraiwesh et al., 2012; H. Li et al., 2010).

### 4.3 MiRNAs orchestrating PSMs productions through TFs

**Figure 4:**
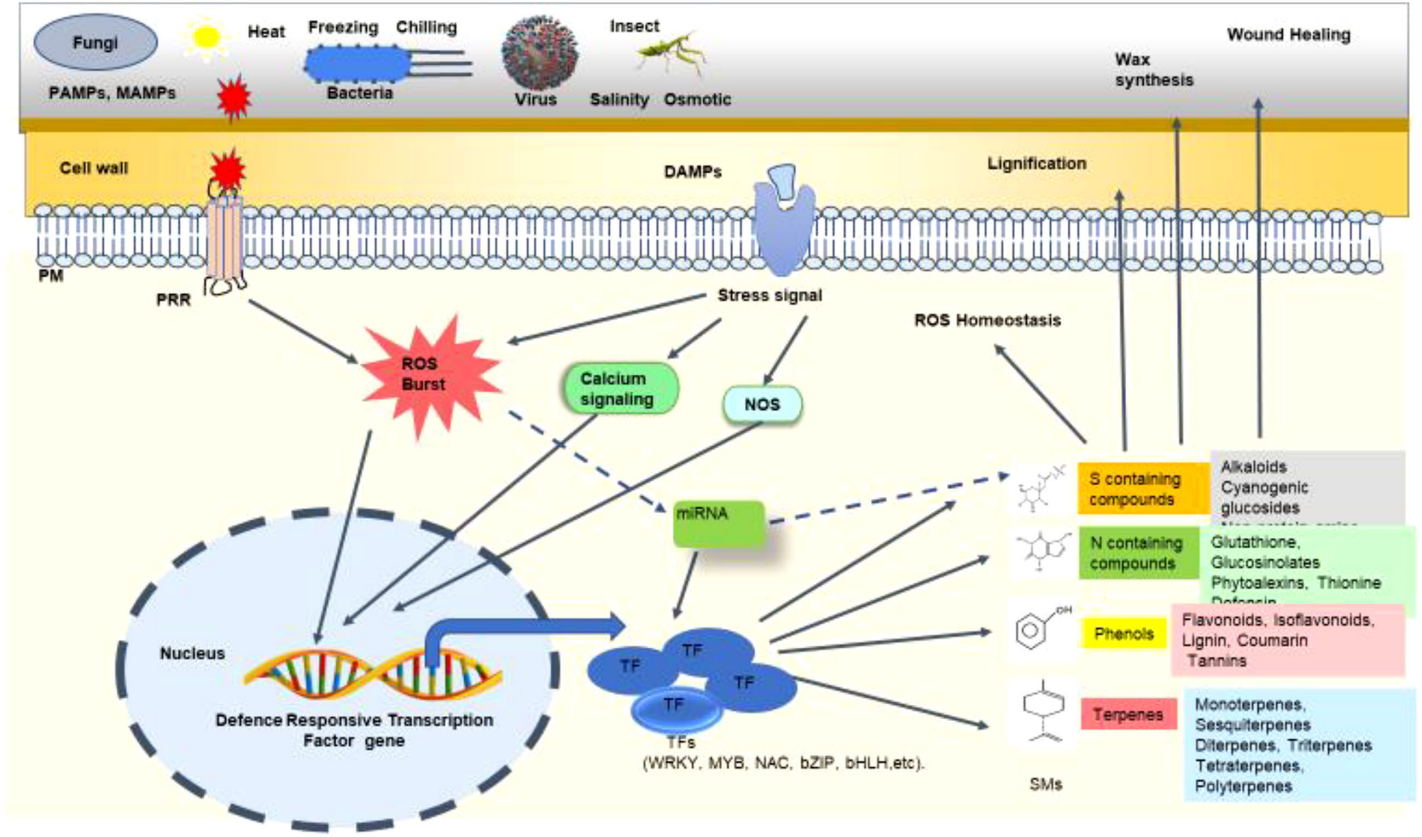
Schematic representation of the mechanism of transcription factors (TFs) regulating secondary metabolites (SMs) biosynthesis under biotic stress. At the time of biotic stress such as fungal, bacterial, viral, or insect attack, various pathogen-associated molecular patterns (PAMPs) and damage-associated molecular patterns (DAMPs) are recognized by the cell wall and membrane receptors like pattern recognition receptors (PRRs). Upon recognition, various stress signals are induced in the form of reactive oxygen species (ROS) burst, nitric oxide (NO) signalling, calcium signalling, etc. These stress signals ultimately trigger the expression of defense-responsive TF genes. These TFs like WRKY, MYB, NAC, bZIP, bHLH, etc. regulate the production of different secondary metabolites in the plant. These SMs protect the plant through lignification, wax synthesis, wound healing, ROS homeostasis, and many other mechanisms. Certain microRNAs (miRNAs) are also involved in controlling TF expression which in turn regulates SM production. The dashed line indicates the exact mechanism of SM production governed by miRNA, which is yet to be deciphered.

Since our focus is to deliberate on regulating PSMs biosynthesis by TFs, it is worthwhile to include how miRNAs modulate PSMs biosynthesis. During any biotic stress, many of the secondary metabolites are differentially regulated. Several miRNAs have been reported to target transcription factors to control secondary metabolite biosynthetic pathways (**Figure 4**) (Jiang et al., 2021). Xia et al., 2012 reported that miR828 targets MYBs to control anthocyanin production. Similarly, miR858-targeted MYBs were associated with lignification, anthocyanin production, and stress responses (Xia et al., 2012). These lignification and anthocyanin production are a well-known mechanisms for wall fortification during pathogen attacks (Shah and Smith, 2020). AtMYB111, AtMYB11, and AtMYB12, transcriptional activators of genes associated with flavonoid biosynthesis, are negatively regulated by miR858. AtMYB111, AtMYB11, and AtMYB12 positively modulated the defense response against *Plectosphaerella cucumerina* in *Arabidopsis* (Camargo Rairez et al., 2017).

Khan et al., 2020 have also demonstrated the interaction of miR858 with MYB1 and concluded that miRNA mimic technique could enhance resistivity against fungal attacks in *Artemisia annua*. The geometrid *Ectropis oblique* poses a significant threat to the tea plant *Camellia sinensis* L. (Wang et al., 2014). During *E. oblique* attack, can-miR171 was reported to interact with bHLH, ultimately contributing to the production of secondary metabolites related to defense (Jeyaraj et al., 2017).

Although an elaborated study has been done in understanding the role of miRNA that regulates transcription factors, there are a few literature that supports the role of miRNA in regulating PSMs. But a correlation of miRNAs regulating TFs that regulate SM production during biotic stress is focused in the present review, which will aid in administering the production of SMs by controlling either the pathway directly or *via* the TFs. Thus, combining the molecular research to industrial engineering opens a whole bunch of opportunity to future researchers and industrialists that use secondary metabolites in production of various products such as medicines and cosmetics.

## 5 Conclusion and future perspective

Since plants are sessile, they have evolved complex regulatory mechanisms to respond to environmental stresses. In topical years, there has been a substantial upsurge in the importance of metabolic adaptation in plants under hostile environmental conditions. PSMs are natural products produced in plants when exposed to potential enemies. There are many transcription factors, WRKY, bHLH, bZIP, NAC, MYB, and AP2/ERF, that mediate the production and regulation of secondary metabolites related to plant defense against pests and pathogen attacks. Therefore, it is crucial to focus on the regulators such as miRNAs that control TFs and understanding their defensive role in plants under biotic constraints. TFs play a role in regulating PSMs fine-tune the expression of these TFs, and miRNas can, synchronize the synthesis and functional regulation of SMs during any pathogenic attack. This information can be utilized in future to modulate the production of SMs in any desired crop creating an enhanced variety in terms of resistivity, medicinal value or yield. In future, specific technologies, like miRNA mimicking, can be widely used to develop advanced SMs-producing plants to aid crops to combat stress and also to the pharmaceutical industries to produce desired amounts of medicinally important SMs. Since ancient times, plant extracts from a diverse range of medicinal plants have been used in the treatment of different diseases, owing to the presence of specific PSMs. With the recent increase in inclination towards traditional medicine (Chinese medicine and Ayurveda) and plant products for treating various diseases like diabetes, respiratory problems, arthritis, malaria and even certain cancers, the research on PSMs has gained momentum (Seca and Pinto, 2019). A thorough understanding of molecular pathways governing SM production can thus aid in enhanced SM producing engineered plants.

## Author contributions

AS and IS conceptualized and supervised the study. AS, MK, IS, and AR contributed to the investigation. MK wrote the original draft. AS, IS, and AR contributed to reviewing, editing, and visualization. AS, IS, and AR contributed to the formal analysis. AS, IS, and AR contributed to funding acquisition. AS and IS contributed to resources. All authors contributed to the article and approved the submitted version.
